# Degradation of the In-plane Shear Modulus of Structural BFRP Laminates Due to High Temperature

**DOI:** 10.3390/s18103361

**Published:** 2018-10-08

**Authors:** Yu-Jia Hu, Cheng Jiang, Wei Liu, Qian-Qian Yu, Yun-Lai Zhou

**Affiliations:** 1 School of Mechanical Engineering, University of Shanghai for Science and Technology, Shanghai 200093, China; huyujia@126.com (Y.-J.H.); 18721287532@163.com (W.L.); 2 Department of Civil and Environmental Engineering, The Hong Kong Polytechnic University, Hong Kong SAR, China; 3 Department of Structural Engineering, Tongji University, No. 1239 Siping Road, Shanghai 200092, China; qianqian.yu@tongji.edu.cn; 4 Department of Civil and Environmental Engineering, National University of Singapore, Singapore 119077, Singapore; ceezyl@nus.edu.sg

**Keywords:** basalt fiber reinforced polymer (BFRP), digital image correlation (DIC) sensor, in-plane shear modulus, high-temperature test, thermal behavior

## Abstract

The behavior of fiber reinforced polymer (FRP) composites at high temperature is a critical issue that needs to be clearly understood for their structural uses in civil engineering. However, due to technical difficulties during testing at high temperature, limited experimental investigations have been conducted regarding the thermal behavior of basalt fiber reinforced polymer (BFRP) composites, especially for the in-plane shear modulus of BFRP laminates. To this end, both an analytical derivation and an experimental program were carried out in this work to study the in-plane shear modulus of BFRP laminates. After the analytical derivation, the in-plane shear modulus was investigated as a function of the elastic modulus in different directions (0°, 45° and 90° of the load-to-fiber angle) and Poisson’s ratio in the fiber direction. To obtain the in-plane shear modulus, the four parameters were tested at different temperatures from 20 to 250 °C. A novel non-contacting digital image correlation (DIC) sensing system was adopted in the high-temperature tests to measure the local strain field on the FRP samples. Based on the test results, it was found that the elastic moduli in different directions were reduced to a very low level (less than 20%) from 20 to 250 °C. Furthermore, the in-plane shear modulus of BFRP at 250 °C was only 3% of that at 20 °C.

## 1. Introduction

Advanced fiber reinforced polymer (FRP) composite materials have been widely used in many areas such as mechanical uses [1,2,3] and structural rehabilitation in civil engineering [4,5,6,7,8]. Recently, as the corrosion issue highly decreases the mechanical behavior of steel reinforced concrete (RC) structures in moist and corrosive environments [9], FRPs have offered an environmentally friendly option by replacing steel in the new structures [10]. Structural FRP (refers to FRP for structural uses in civil engineering [11]) composite material is made of a polymer matrix reinforced with different types of fibers. Therefore, FRP composites can be classified by the type of fibers such as carbon fiber reinforced polymer (CFRP), glass fiber reinforced polymer (GFRP), and aramid fiber reinforced polymer (AFRP). Recently, the newly developed basalt fiber reinforced polymer (BFRP) has attained increasing applications due to its high performance and low cost [12,13,14,15]. Most investigations regarding FRP applications have focused on the behaviors of FRP products [16,17,18,19] or FRP-involved composite structures [20,21,22,23] under normal temperature conditions. However, it is known that FRPs have limitations regarding high-temperature or fire resistance, and the investigations on the thermal behaviors of FRP are critical and still need to be further studied. 

Bai et al. [24] have proposed a theoretical model for the stiffness of GFRPs at a high temperature based on the storage modulus results of dynamic mechanical analysis (DMA) tests from −40 to 250 °C. DMA is a common technique used to study the mechanical characteristics and thermal behavior, such as the glass transition temperature, of polymer materials. Chowdhury et al. [25] used 2-D particle image velocimetry (PIV) technology to measure the strain field on the GFRP samples in a range of 20 to 200 °C. These authors found that the tensile strength of the GFRP at 200 °C retained 40% of that at room temperature. During their high-temperature tests, the thermal chamber was not kept under vacuum, and the results of Poisson’s ratio were not reported. The effect of the loading rate on the tension properties of the GFRP laminates under various temperatures (from 25 to 110 °C) was carried out recently by Mahato et al. [26]. Similar studies regarding the temperature effects have been conducted on CFRPs [27,28,29,30] and AFRPs [31].

Recently, BFRP composites have been widely investigated as an environmentally friendly and cost-effective alternative of the other types of FRPs due to good tensile properties and satisfactory resistance to chemical attack or fire with less poisonous fumes [32]. Basalt fibers are obtained by a melting process from basalt rocks. The different chemical elements in basalt rocks (such as ferric ions) can cause different thermal behaviors, which need further investigations. To this end, Lu et al. [33,34] investigated the effect of thermal aging on the behaviors of the BFRP plates. The BFRP plates were exposed to different temperatures in an oven for four hours before cooling down and testing. The thermal aging results in the decomposition of the epoxy resin matrix, which leads to fiber debonding [33,34]. On the other hand, Lu et al. [33,35] studied the tensile properties of BFRP plates at high testing temperatures. However, the strain data is obtained by an extensometer under elevated temperatures, which can provide the strain data at one location and in one direction.

Another important factor that influences the environmental performance of FRP composites is polymer matrix [36]. In the existing literature, apart from DMA tests [24,37,38], multiscale simulations [39,40] and nano-scale digital image correlation (DIC) technology [40,41,42] are adopted to model the mechanical degradation and measure the deformation/cracking of the polymer matrix, respectively. It was found that the matrix significantly governs the thermal mechanical behaviors, especially at a temperature that is higher than the glass transition temperature of the polymer matrix. 

In summary, all the existing research regarding the thermal behavior of FRP materials has studied the mechanical properties, such as tensile modulus and tensile strength, which can be directly tested. The in-plane shear modulus, which is an important mechanical parameter in composite materials [43], has not been studied so far, largely due to the complexities of the test and the analysis. In the practical engineering applications, the stress conditions of the applied FRP material cannot be under ideal states, due to many unavoidable factors such as load eccentricity and construction errors. FRP may be subjected to not only tension forces, but also shear forces. Therefore, shear modulus of FRP laminate is one of the key factors in the accurate analysis which has been often ignored.

This paper aims to investigate, for the first time, the thermal effect on the in-plane shear modulus of BFRP laminates for structural uses in civil engineering. To derive the in-plane shear modulus, the elastic modulus in three different directions, as well as Poisson’s ratio, were obtained using the newly developed testing instrumentation with a DIC sensing system. This sensing system was firstly used in the composite material testing. After this work, the thermal behavior of FRP can be understood more clearly.

## 2. Derivations of the In-plane Shear Modulus

### 2.1. E_1_, E_2_, and ν_21_

**Figure 1:**
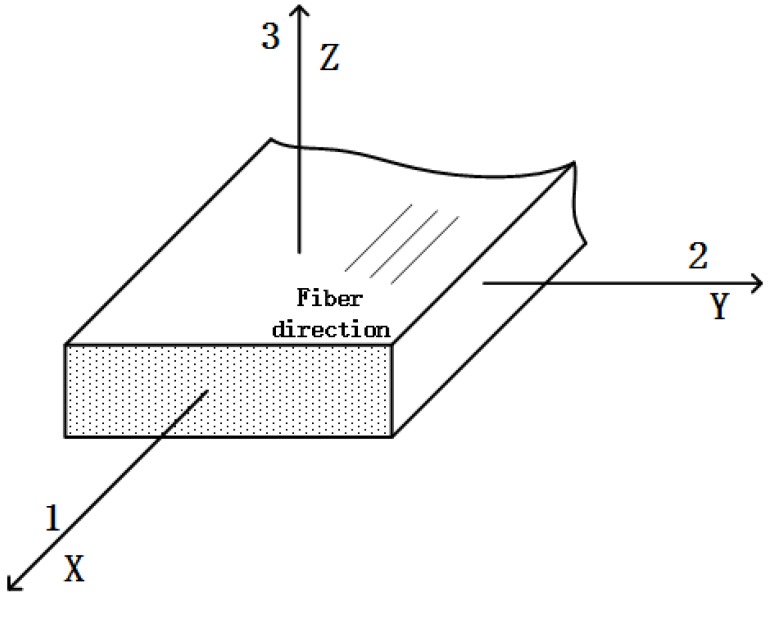
The schematic diagram of the BFRP plate.

**Figure 2:**
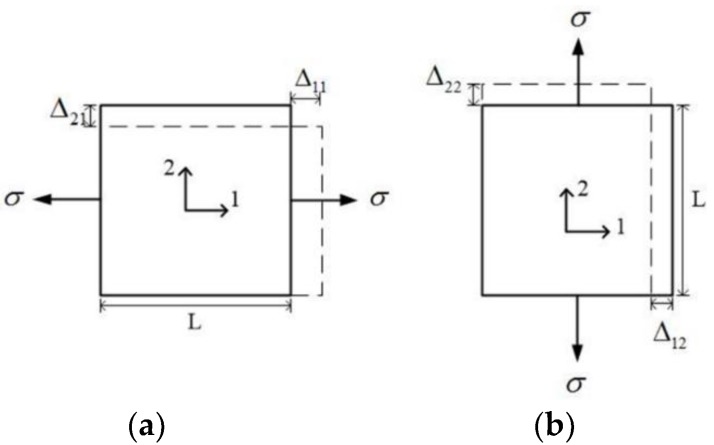
Deformation in plane 1–2. (**a**) Loading on axis 1, (**b**) Loading on axis 2.

The FRP laminates for structural uses in civil engineering are usually unidirectional, e.g., [44,45,46,47]. The unidirectional structural BFRP plate is generally a certain type of anisotropic material known as especially orthotropic material and can be considered as transversely isotropic material [48,49,50,51,52], as shown in Figure 1. In addition, Sections 2–3 in Figure 1 can be considered to have anisotropic material. When analyzing the micromechanical behaviors, the thickness (direction 3 in Figure 1) is quite small compared with the other two directions (directions 1 and 2 in Figure 1). Hence, the plate under tension can be subjected to the plane stress state (i.e., *σ*_3_ = *τ*_23_ = *τ*_31_ = 0). The stress-strain relationship in the plane stress state is

(1) $$\left[\begin{array}{l} \sigma_{1}\\ \sigma_{2}\\ \tau_{12} \end{array}\right]=\left[\begin{array}{lll} Q_{11} & Q_{12} & 0\\ Q_{12} & Q_{22} & 0\\ 0 & 0 & Q_{33} \end{array}\right]\left[\begin{array}{l} \varepsilon_{1}\\ \varepsilon_{2}\\ \gamma_{12} \end{array}\right]$$

where *σ_i_* and *ε_i_* are the stress and strain in direction *i*, respectively; *τ_ij_* and *γ_ij_* are shear stress and shear strain in *i*-*j* plane; *Q_ij_* is the elastic constant given by

(2a) $$Q_{11}=\frac{E_{1}}{1-\nu_{12}\nu_{21}}$$

(2b) $$Q_{22}=\frac{E_{2}}{1-\nu_{12}\nu_{21}}$$

(2c) $$Q_{12}=\frac{\nu_{21}E_{2}}{1-\nu_{12}\nu_{21}}=\frac{\nu_{12}E_{1}}{1-\nu_{12}\nu_{21}}$$

(2d) $$Q_{33}{=G}_{12}$$

in which *E*_1_, *E*_2_ and *E*_3_ are the elastic moduli in directions 1, 2 and 3, respectively; *G*_12_ is the in-plane shear modulus in plane 1–2; and *ν*_21_ and *ν*_12_ are the values of Poisson’s ratio in the two directions, as shown in Figure 2. When normal stress is applied in direction 1 (Figure 2a), the strain values can be calculated as

(3a) $$\varepsilon_{1}=\frac{\sigma }{E_{1}}=\frac{\Delta_{11}}{L}$$

(3b) $$\varepsilon_{2}=\left(-\frac{\nu_{21}}{E_{1}}\right)\sigma =-\frac{\Delta_{21}}{L}$$

On the other hand, the strains for the specimen subjected to a normal load at direction 2 (Figure 2b) can be calculated as

(4a) $${\varepsilon_{2}}^{\prime }=\frac{\sigma }{E_{2}}=\frac{\Delta_{22}}{L}$$

(4b) $${\varepsilon_{1}}^{\prime }=\left(-\frac{\nu_{12}}{E_{2}}\right)\sigma =-\frac{\Delta_{12}}{L}$$

As Δ_12_ = Δ_21_ for the same *σ*, which means the deformation in direction 2 in Figure 2a is equal to the deformation in direction 1 in Figure 2b, it can be concluded that

(5) $$\frac{\nu_{21}}{E_{1}}=\frac{\nu_{12}}{E_{2}}$$

Hence, there are in total four independent elastic parameters (i.e., *E*_1_, *E*_2_, *ν*_21_, and *G*_12_) in the plane stress problem of the orthotropic thin plates. Parameters *E*_1_, *E*_2_, and *ν*_21_ can be directly obtained from tension tests. In addition, *G*_12_ can be calculated from the derivations by the other three parameters, which are discussed in the following section.

### 2.2. Shear Modulus G_12_ by the 45° Off-Axis Tension Tests

**Figure 3:**
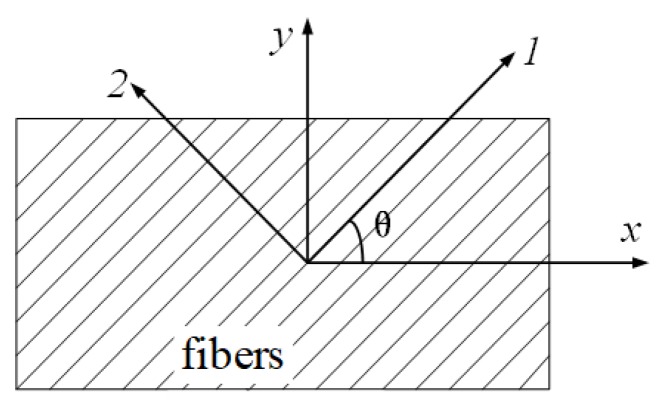
Schematic diagram of the off-axis tension tests.

As the BFRP plate for structural uses is one type of orthotropy material, the coupling effect will cause a shear deformation when it is subjected to the asymmetric off-axis loading. A 45° off-axis tension test is an experimental method for the analysis of the shear stress field on the oblique section. It has been widely adopted to measure the shear modulus of the orthotropy material due to the ease of specimen preparation and load application. The schematic diagram of the off-axis tension test is shown in Figure 3. When the specimen is under a state of plane stress, the strain components in the *x-y* coordinate system of the laminate are *ε_x_*, *ε_y_* and *γ_xy_*. The fiber direction (direction 1) has an angle of *θ* with the *x*-axis, as shown in Figure 3. The strain components in the 1–2 coordinate system (Figure 3) are *ε*_1_, *ε*_2_ and *γ*_12_. For the laminate element with *dl* of its diagonal length (in fiber direction), an increase in the diagonal length can be calculated as

(6a) $$\varepsilon_{1}dl=\varepsilon_{x}dx\cos\theta +\varepsilon_{y}dy\sin\theta +\gamma_{xy}dy\cos\theta$$

As dx=dlcosθ and dy=dlcosθ, Equation (6a) can be rewritten as

(6b) $$\varepsilon_{1}=\varepsilon_{x}\cos^{2}\theta +\varepsilon_{y}\sin^{2}\theta +\gamma_{xy}\sin\theta \cos\theta$$

Similarly, Equations (7) and (8) can be expressed for *ε*_2_ and *γ*_12_

(7) $$\varepsilon_{2}=\varepsilon_{x}\sin^{2}\theta +\varepsilon_{y}\cos^{2}\theta -\gamma_{xy}\sin\theta \cos\theta$$

(8) $$\gamma_{12}=-2\varepsilon_{x}\sin\theta \cos\theta +2\varepsilon_{y}\sin\theta \cos\theta +\gamma_{xy}(\cos^{2}\theta -\sin^{2}\theta )$$

Equations (6)–(8) can be rewritten to be the strain formula of the rotated axes:

(9) $$\left[\begin{array}{l} \varepsilon_{1}\\ \varepsilon_{2}\\ \gamma_{12} \end{array}\right]=\left[\begin{array}{l} \begin{array}{ccc} \cos^{2}\theta & \sin^{2}\theta & \sin\theta \cos\theta \end{array}\\ \begin{array}{ccc} \sin^{2}\theta & \cos^{2}\theta & -\sin\theta \cos\theta \end{array}\\ \begin{array}{cc} -2\sin\theta \cos\theta & \begin{array}{cc} 2\sin\theta \cos\theta & \cos^{2}\theta -\sin^{2}\theta \end{array} \end{array} \end{array}\right]\left[\begin{array}{l} \varepsilon_{x}\\ \varepsilon_{y}\\ \gamma_{xy} \end{array}\right]$$

Similarly, the stress components in the 1–2 coordinate system can be calculated by

(10) $$\left[\begin{array}{l} \sigma_{1}\\ \sigma_{2}\\ \tau_{12} \end{array}\right]=\left[\begin{array}{l} \begin{array}{ccc} \cos^{2}\theta & \sin^{2}\theta & 2\sin\theta \cos\theta \end{array}\\ \begin{array}{ccc} \sin^{2}\theta & \cos^{2}\theta & -2\sin\theta \cos\theta \end{array}\\ \begin{array}{cc} -\sin\theta \cos\theta & \begin{array}{cc} \sin\theta \cos\theta & \cos^{2}\theta -\sin^{2}\theta \end{array} \end{array} \end{array}\right]\left[\begin{array}{l} \sigma_{x}\\ \sigma_{y}\\ \tau_{xy} \end{array}\right]$$

When calculating the strain values by stress data, the following formula can be obtained:

(11) $$\left[\begin{array}{l} \varepsilon_{1}\\ \varepsilon_{2}\\ \gamma_{12} \end{array}\right]=\left[\begin{array}{lll} S_{11} & S_{12} & 0\\ S_{12} & S_{22} & 0\\ 0 & 0 & S_{66} \end{array}\right]\left[\begin{array}{l} \sigma_{1}\\ \sigma_{2}\\ \tau_{12} \end{array}\right]$$

where $S_{11}=\frac{1}{E_{1}}$, $S_{12}=-\frac{\nu_{21}}{E_{1}}$, $S_{22}=\frac{1}{E_{2}}$, $S_{66}=\frac{1}{G_{12}}$.

For the *x-y* coordinate system:

(12a) $$\left[\begin{array}{l} \varepsilon_{x}\\ \varepsilon_{y}\\ \gamma_{xy} \end{array}\right]=\left[\begin{array}{l} \begin{array}{ccc} {S_{11}}^{\prime } & {S_{12}}^{\prime } & {S_{16}}^{\prime } \end{array}\\ \begin{array}{ccc} {S_{12}}^{\prime } & {S_{22}}^{\prime } & {S_{26}}^{\prime } \end{array}\\ \begin{array}{ccc} {S_{16}}^{\prime } & {S_{26}}^{\prime } & {S_{66}}^{\prime } \end{array} \end{array}\right]\left[\begin{array}{l} \sigma_{x}\\ \sigma_{y}\\ \tau_{xy} \end{array}\right]$$

where

(12b) $$\begin{array}{ll} \frac{1}{E_{x}} & ={S_{11}}^{\prime }=S_{11}\cos^{4}\theta +(2S_{12}+S_{66})\sin^{2}\theta \cos^{2}\theta +S_{22}\sin^{4}\theta \\ & =\frac{1}{E_{1}}\cos^{4}\theta +(\frac{1}{G_{12}}-\frac{2\nu_{21}}{E_{1}})\sin^{2}\theta \cos^{2}\theta +\frac{1}{E_{2}}\sin^{4}\theta \end{array}$$

(12c) $$\begin{array}{ll} \frac{-\nu_{xy}}{E_{x}} & ={S_{12}}^{\prime }=S_{12}(\sin^{4}\theta +\cos^{4}\theta )+(S_{11}+S_{22}-S_{66})\sin^{2}\theta \cos^{2}\theta \\ & =\frac{-\nu_{21}}{E_{1}}(\sin^{4}\theta +\cos^{4}\theta )+(\frac{1}{E_{1}}+\frac{1}{E_{2}}-\frac{1}{G_{12}})\sin^{2}\theta \cos^{2}\theta \end{array}$$

(12d) $$\begin{array}{ll} \frac{1}{G_{xy}} & ={S_{66}}^{\prime }=4(S_{11}+S_{22}-2S_{12}-\frac{1}{2}S_{66})\sin^{2}\theta \cos^{2}\theta +S_{66}(\sin^{4}\theta +\cos^{4}\theta )\\ & =4(\frac{1+2\nu_{21}}{E_{1}}+\frac{1}{E_{2}}-\frac{1}{2G_{12}})\sin^{2}\theta \cos^{2}\theta +\frac{1}{G_{12}}(\sin^{4}\theta +\cos^{4}\theta ) \end{array}$$

For the case of the 45° off-axis tension test, *θ* = 45°, $\sigma_{y}=\tau_{xy}=0$, and $\frac{1}{E_{x}}=\frac{1}{E_{1}}\cos^{4}\theta +(\frac{1}{G_{12}}-\frac{2\nu_{21}}{E_{1}})\sin^{2}\theta \cos^{2}\theta +\frac{1}{E_{2}}\sin^{4}\theta$; therefore,

(13a) $$\frac{1}{E_{45}}=\frac{\varepsilon_{x}}{\sigma_{x}}=\frac{1}{4}\left[\frac{1}{E_{1}}+\left(\frac{1}{G_{12}}-\frac{2\nu_{21}}{E_{1}}\right)+\frac{1}{E_{2}}\right]$$

(13b) $$G_{12}=\frac{1}{\frac{4}{E_{45}}-\frac{1}{E_{1}}-\frac{1}{E_{2}}+\frac{2\nu_{21}}{E_{1}}}$$

## 3. Experimental Program

Based on the derivations above, the in-plane shear modulus *G*_12_ can be obtained when *E*_1_, *E*_2_, *ν*_21_ and *E*_45_ are known. And, the values of *E*_1_, *E*_2_, *ν*_21_ and *E*_45_ can be obtained by testing at different temperatures. The experimental program was presented in detail in the following sections.

### 3.1. Instrumentation and Measuring System for the High-Temperature Tests

**Figure 4:**
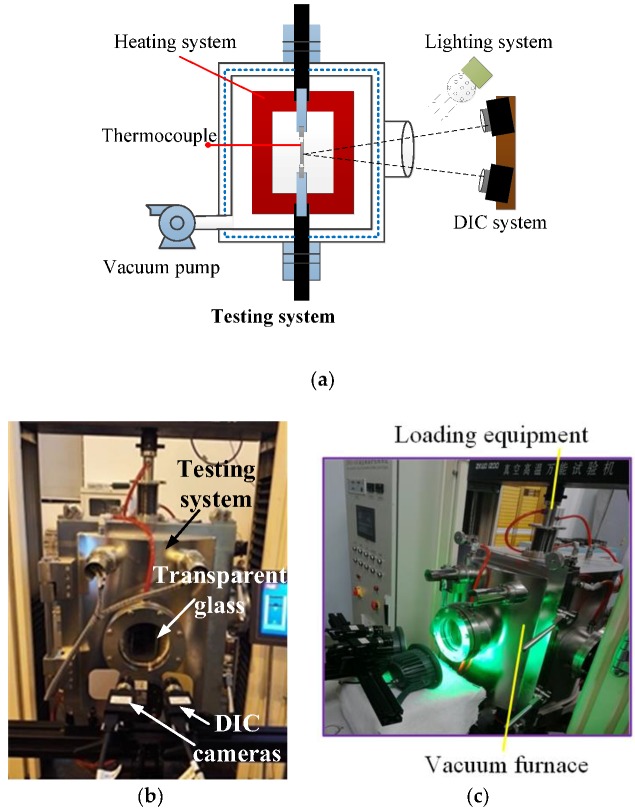
High-temperature testing and measuring system. (**a**) Test setup, (**b**) DIC sensing system, (**c**) lighting system.

The high-temperature testing and measuring system (Figure 4), which was recently developed by the authors, consists of four subsystems: (1) a universal testing machine located in a vacuum chamber; (2) a heating system including a high-temperature vacuum pump and a high-temperature vacuum furnace with associated measuring sensors; (3) a stable lighting system with multiple green LED illuminators; and (4) a 3-D DIC sensing system (Figure 4b) including two symmetrically placed high-megapixel digital cameras and a computer. The cameras were equipped with 532 nm narrow-band filters to overcome the difficulties of surface radiation due to the high temperature and to block out ambient light. Lighting conditions also affect the quality of the speckle pattern. In the present work, a lighting system with multiple green LED illuminators and two 532 nm narrow band (10 nm) filters to block out ambient light are used to obtain the high contrast speckle pattern (Figure 4c). An image acquisition and analysis software installed on a computer was used to obtain the deformation and strain fields. Unlike the other high-temperature tests on FRP composites in the literature, the samples in this work were tested in a vacuum environment, which can provide more reliable results by avoiding the unpredicted material oxidation at high temperature. The vacuum environment also can avoid the optical refraction from hot air to room-temperature air, which can lead to inaccurate measuring results by DIC.

### 3.2. Specimen Design and Preparation

**Figure 5:**
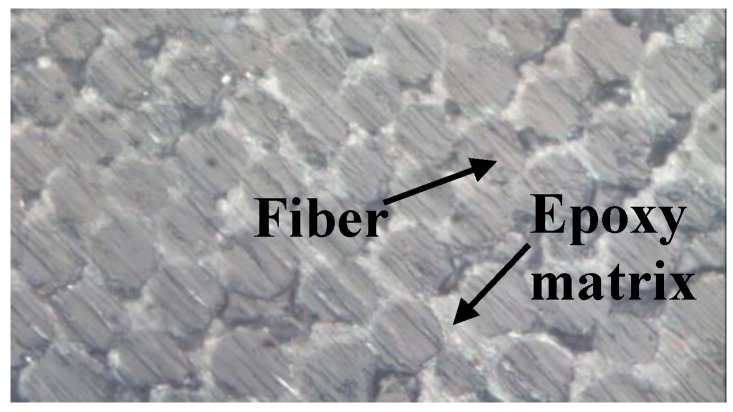
Typical microscope image of a BFRP laminate section.

**Figure 6:**
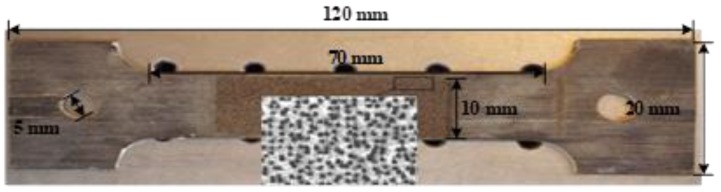
Tensile coupon specimen.

The unidirectional BFRP laminates used in this study, provided by Jiangsu Green Materials Vally New Material T&D Co., Ltd. (GMV) (Nanjing, China), had a thickness of 2 mm. The polymer matrix in this composite product was an epoxy 9804 A/B from Bluestar Wuxi Petrochemical Co, Ltd. (Wuxi, China) with 3.6 GPa, 142 N/m, 3.8, and 1.69 KJ/m for elastic modulus, fracture toughness, dielectric constant and impact strength respectively. The tested glass transition temperature of the matrix was equal to 92 °C approximately by DMA testing [53]. The fiber volume fraction of the BFRP laminates used in this work was estimated to be 56.3%. The BFRP laminates were manufactured by a pultrusion process, which leads to a very limited void fraction (as shown in Figure 5 from microscope image analysis) that can be ignored in analysis. The size of the tensile coupons was prepared according to the testing standard [54] and testing equipment, as shown in Figure 6. The specimens were designed to be tested under four different temperatures (i.e., 20 °C, 100 °C, 200 °C and 250 °C). Three different angles between loading direction and fiber direction (i.e., 0°, 45° and 90°) were investigated. Two nominally identical specimens (named 1st and 2nd in Table 1) were tested for each design, which was less than the common number of three or five but can be considered acceptable in the literature for FRP applications [55,56,57,58,59,60]. Therefore, in total, 4 (temperature levels) × 3 (loading angles) × 2 (identical specimens) = 24 specimens were carried out for tension testing in this work. The details of the tested specimens can be found in Table 1. The specimens with 0° and 90° of the load-to-fiber angle were carried out for measuring the values of *E*_1_, *E*_2_ and *ν*_21_, which are discussed in Section 2.1. The specimens with 45° of the load-to-fiber angle were prepared for the off-axis tension tests to derive the in-plane shear modulus, which is investigated in Section 2.2.

**Figure 7:**
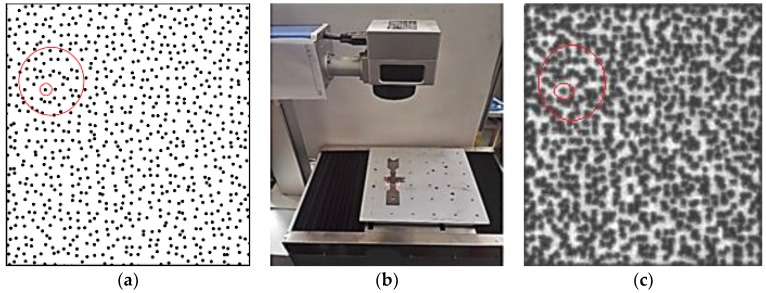
Laser engraving machine and engraved speckles: (**a**) computer-generated speckle pattern, (**b**) laser engraving system, and (**c**) laser engraved speckle pattern based on the computer input.

In the DIC sensing and measuring system, the specimen surfaces are usually treated by “added-on” speckle patterns such as random spraying [61,62,63,64,65,66,67]. However, such “added-on” speckle patterns tend to fail before the specimens reach the intended high temperature. To solve this problem, an engraving technique was recently developed by the authors to produce random speckle patterns directly onto the specimen surfaces [68]. The speckle patterns of various speckle sizes, densities and distributions can be generated by a specially designed fiber laser engraving system without damaging the material, as shown in Figure 7. The similarities in the red circles in Figure 7 show the consistency between the two speckling methods. The technique developed consists of the following steps:

(a) Generate random two-dimensional coordinates of points in the X-Y plane. The density and distribution of the speckles can be controlled by computer.
(b) Assign a radius to each speckle point, and the size of the speckles can be controlled by computer.
(c) Generate a computer-generated speckle pattern by using the two-dimensional coordinates of random points and the radius of the speckles.
(d) Import the computer-generated speckle pattern into the specially designed fiber laser engraving system.
(e) By adjusting the output power and exposure time of the fiber laser engraving system, a laser-engraved speckle pattern can be created. The depth of the speckle pattern can be controlled by computer.

**Figure 8:**
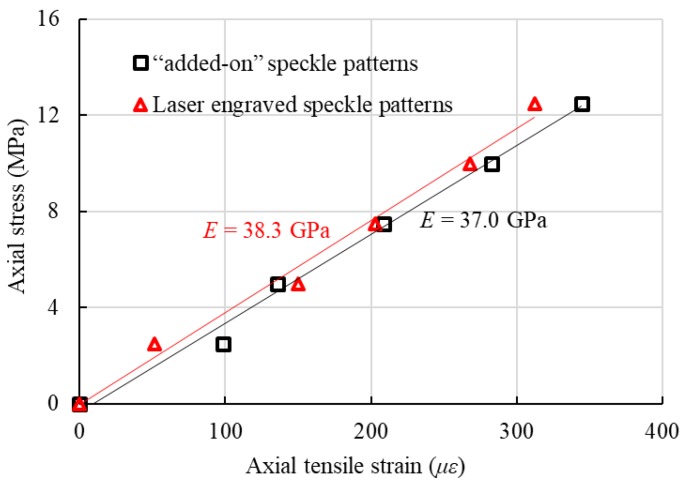
Test results for the BFRP samples with different speckle types.

Therefore, the size, density and depth of the speckles can be controlled to suit a particular situation. And the pattern will never disappear until specimen failure because it is a part of the specimen. Before testing, trial tests for calibrating different speckle types (i.e., laser engraving and spraying) at room temperature were conducted. The small error on the achieved elastic modulus (approximately 3%) in Figure 8 indicates the reliability of the proposed laser engraving method. Using this testing and measuring system, the testing temperature can reach as high as 2000 °C, which can be suitable for high-temperature tests on most types of materials.

### 3.3. Testing

**Figure 9:**
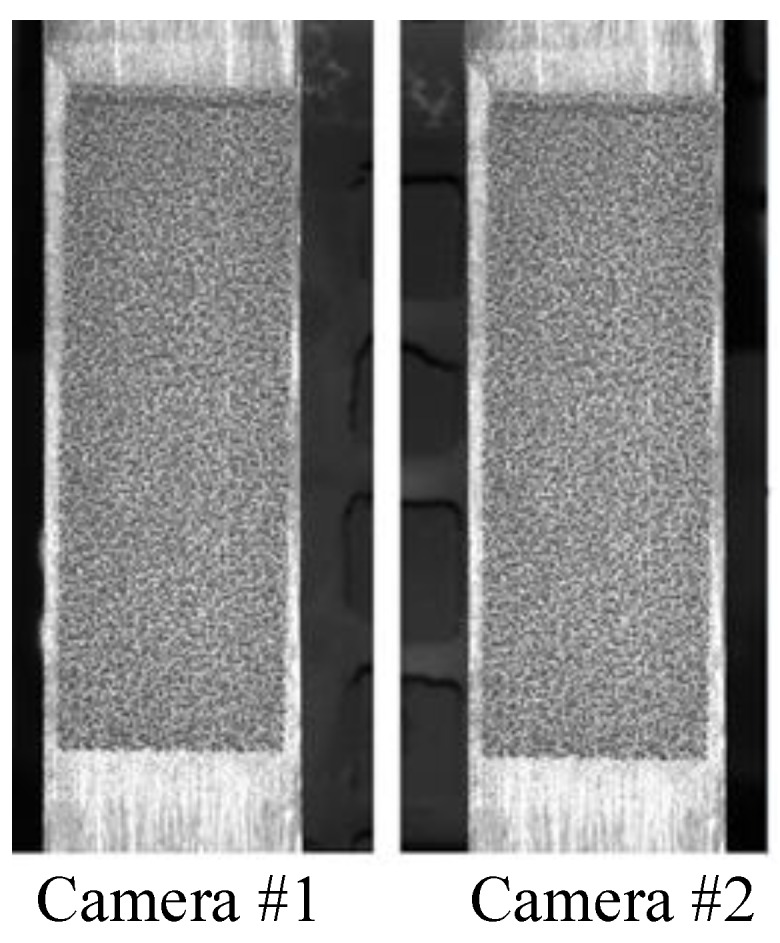
Captured DIC images when testing.

All the BFRP specimens were loaded stepwise in uniaxial tension under a displacement control of 0.1 mm/min. The gradient of heating the sample during the tests was approximately 20 °C/min. During testing, the sample surface was illuminated by a high-power LED light through a quartz glass. Two CCD cameras with 2048 × 2048 pixel resolution and two Schneider Xenoplan lenses of 1.9/35 mm were used to record the deformation field of the speckle pattern. Before applying a load, the specimen in the vacuum room was heated to the target temperature, and then the temperature was maintained for four minutes. The specimens were preloaded with 300 N and unloaded several times before loading. Because the clamping equipment in the high-temperature testing system is an interlocking type, the BFRP sample fails at the specimen ends by shear failure before the FRP rupture. This clamping method can be accepted because the aim of this work is to investigate the in-plane moduli from elastic modulus and Poisson’s ratio of the BFRP at different temperatures. The ultimate stress and strain of the BFRP at high temperature, which have been studied by others [33,35], were not necessary for the methodology in this work. Typical captured DIC images are illustrated in Figure 9.

## 4. Test Results and Discussion

**Figure 10:**
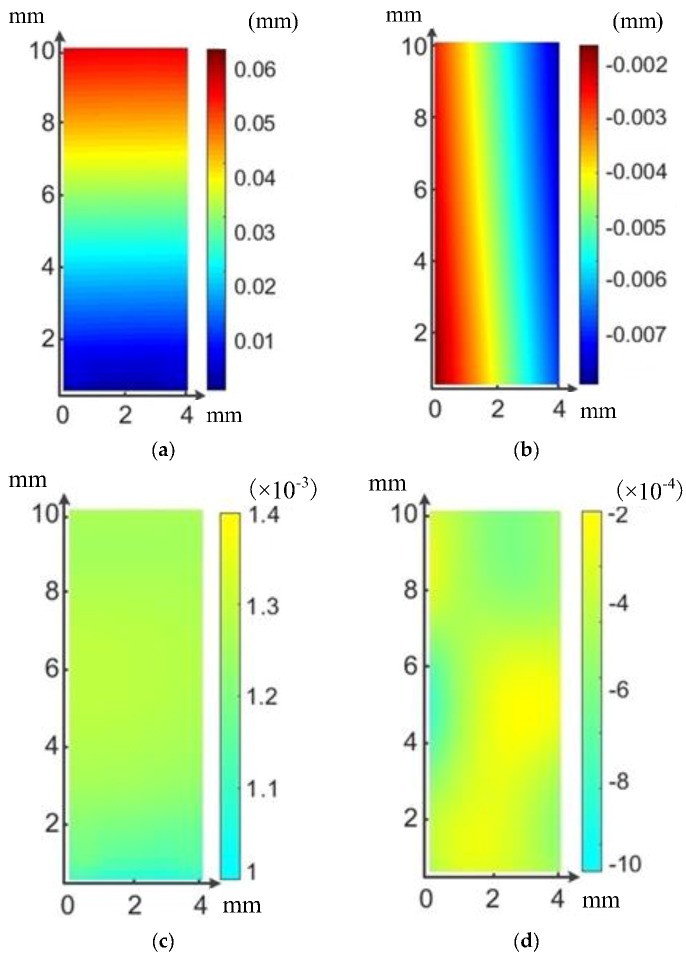
Typical DIC results at a tensile load of 300 N and 250 °C. (**a**) Vertical displacement field (fiber direction), (**b**) Horizontal displacement field (perpendicular to the fiber direction), (**c**) Vertical strain field and (**d**) Horizontal strain field.

The strain and displacement fields can be obtained from analyzing by digital signal processing (DSP) software in the DIC system. The precision of the displacement measurement can reach a magnitude of a micrometer. The typical displacement and strain fields in different directions of tested BFRP specimen are shown in Figure 10. 

The width (*b_f_*) and thickness (*t_f_*) of the BFRP specimens are 10 mm and 2 mm, respectively. The tested stress values (*σ*) can be calculated as *σ* = *F*/(*b_f_t_f_*). The strain values were obtained by DIC analysis. The values of elastic moduli were obtained by linear regression from the tested stress and strain values. The values of Poisson’s ratio were obtained from the strain values in two directions.

**Figure 11:**
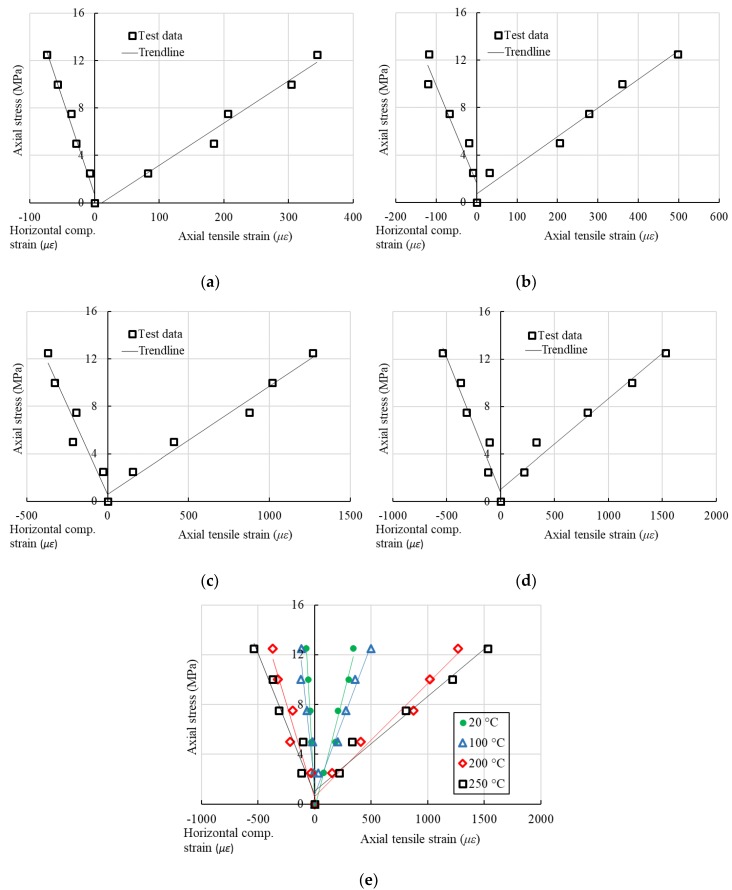
Stress-strain response of the BFRP specimens loaded in the *x*-direction (1st group). (**a**) Test at 20 °C, (**b**) Test at 100 °C, (**c**) Test at 200 °C, (**d**) Test at 250 °C, (**e**) Comparison.

When applying the tensile stress on the BFRP specimens, the fibers were assumed to have the same deformation with the matrix with a linear stress-strain relationship, which fits *σ_k_* = *E_i_ε_j_* (e.g., *σ*_1_ = *E*_1_*ε*_1_ or *σ*_2_ = *E*_2_*ε*_2_). If *σ*_1_ is applied on the specimen only, then the specimen can have deformations in both *x* and *y* directions with *ε*_1_ and *ε*_2_. According to the definition of Poisson’s ratio, the value of Poisson’s ratio can be determined as $\nu_{21}=\left|\varepsilon_{2}/\varepsilon_{1}\right|$. The stress-strain (two directions) responses of the BFRP specimens loaded in the fiber direction are illustrated in Figure 11. The values of *E*_2_ and *E*_45_ are similarly obtained by linear regression analysis from the 90° and 45° tension tests, respectively. All the values of the elastic moduli in different directions and Poisson’s ratios are listed in Table 1.

**Figure 12:**
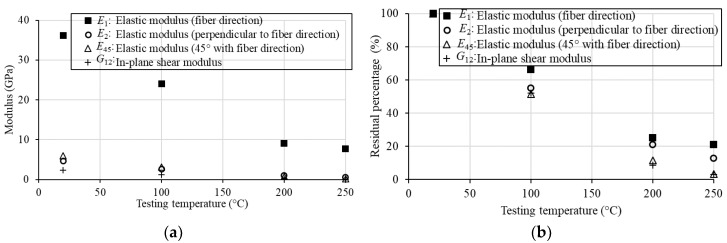
Degradation of the BFRP modulus due to high temperature. (**a**) Values of the different moduli, (**b**) Residual percentages of the different moduli.

The comparison of the stress-strain relationships at different testing temperatures can be found in Figure 11e, which shows the significant degradations with the increasing temperatures. Once the values of *E*_1_, *E*_2_, *ν*_21_ and *E*_45_ are known, the in-plane shear moduli *G*_12_ of the BFRP specimens tested at different temperature levels can be calculated using Equation (13b). The calculated *G*_12_ values, as well as the average values of *E*_1_, *E*_2_, *ν*_21_ and *E*_45_, are listed in Table 2. A significant degradation of the in-plane shear modulus *G*_12_, as well as *E*_1_, *E*_2_, *ν*_21_ and *E*_45_, is illustrated in Figure 12. When the temperature is 100 °C, the in-plane shear modulus decreases to 52.2% of that at 20 °C. In addition, *E*_1_, *E*_2_ and *E*_45_ reduce to 66.3%, 55.3% and 51.7% from 20 to 100 °C, respectively. At 250 °C, *E*_45_ and *G_12_* decrease more rapidly (with 8.7% and 3.0% of those at 20 °C, respectively) than *E*_1_ and *E*_2_. 

As the glass transition temperature of the matrix in the BFRP laminates is 92 °C approximately, the composite materials at this temperature start to have significant degradations. The test results of the specimens tested at 100 °C fit this concept. The degradation due to high temperature is because of the matrix decomposition with increasing temperature. The epoxy resin becomes softer at a higher temperature. Internal debonding occurs in the fibers due to the weak adhesion of epoxy resin matrix at high temperature.

## 5. Conclusions

This paper presents the first study on the degradation of the in-plane shear modulus of structural BFRP laminates due to high temperature. Both analytical and experimental studied were conducted in this work. The following conclusions are drawn from the study:

(1) The derivations illustrated that the in-plane shear modulus can be calculated from four other mechanical parameters (i.e., *E*_1_, *E*_2_, *ν*_21_ and *E*_45_). These four parameters can be directly obtained in an experimental program including both uniaxial tension and off-axis tension tests. The newly developed testing and measuring system with DIC was successfully used to accurately measure the mechanical properties of the BFRP specimens.
(2) The elastic moduli of the BFRP laminates in three directions, *E*_1_, *E*_2_ and *E*_45_, from direct test results, together with the in-plane shear modulus calculated by the analytical formulas, drop to a very low level from 20 to 250 °C. The values of *E*_45_ and *G*_12_ decrease more rapidly (with 8.7% and 3.0% of those at 20 °C, respectively) than *E*_1_ and *E*_2_ at 250 °C.
(3) The degradation of mechanical performances due to high temperature is because of the decomposition of the epoxy resin matrix with increasing temperatures. Internal debonding occurs in the fibers due to the weak adhesion of epoxy resin matrix at high temperature.
(4) The low level of the shear modulus, as well as other mechanical behaviors, of BFRP laminates at high temperatures show the need for the improvement on the thermal behavior for the wide applications using this composite material in the future.

## Figures and Tables

**Table 1:** Details of the tested specimens.

Temperature (°C)	Load-to-Fiber Angle	Specimen ID	*ν*_21_	*E*_1_ (GPa)	*E*_2_ (GPa)	*E*_45_ (GPa)
20	0°	1st	0.215	35.532	-	-
	0°	2nd	0.249	36.955	-	-
	90°	1st	-	-	4.819	-
	90°	2nd	-	-	4.531	-
	45°	1st	-	-	-	5.767
	45°	2nd	-	-	-	6.244
	Standard deviation		0.024	1.006	0.204	0.337
100	0°	1st	0.272	24.148	-	-
	0°	2nd	0.284	23.760	-	-
	90°	1st	-	-	2.778	-
	90°	2nd	-	-	2.358	-
	45°	1st	-	-	-	3.223
	45°	2nd	-	-	-	3.056
	Standard deviation		0.008	0.274	0.297	0.118
200	0°	1st	0.320	9.091	-	-
	0°	2nd	0.309	10.397	-	-
	90°	1st	-	-	0.962	-
	90°	2nd	-	-	1.056	-
	45°	1st	-	-	-	0.683
	45°	2nd	-	-	-	0.770
	Standard deviation		0.008	0.923	0.066	0.062
250	0°	1st	0.335	7. 622	-	-
	0°	2nd	0.324	7. 804	-	-
	90°	1st	-	-	0.639	-
	90°	2nd	-	-	0.604	-
	45°	1st	-	-	-	0.225
	45°	2nd	-	-	-	0.262
	Standard deviation		0.008	0.129	0.025	0.026

**Table 2:** Summary of the test results (average values).

Temperature (°C)	*ν*_21_	*E*_1_ (GPa)	*E*_2_ (GPa)	*E*_45_ (GPa)	*G*_12_ (GPa)
20	0.23	36.2	4.7	6.0	2.3
100	0.28	24.0	2.6	3.1	1.2
200	0.31	9.1	1.0	0.7	0.2
250	0.33	7.7	0.6	0.2	0.07
